# Non-Destructive In Vitro Evaluation of an Internal Adaptation of Recent Pulp-Capping Materials in Permanent Teeth Using OCT and Micro-CT

**DOI:** 10.3390/children10081318

**Published:** 2023-07-31

**Authors:** Ahmed Y. Alzahrani, Amani A. Al Tuwirqi, Nada O. Bamashmous, Turki A. Bakhsh, Eman A. El Ashiry

**Affiliations:** 1 Pediatric Dentistry Department, Faculty of Dentistry, King Abdulaziz University, Jeddah 21589, Saudi Arabia; aalzahrani3670@stu.kau.edu.sa (A.Y.A.); nobamashmous@kau.edu.sa (N.O.B.); eelashiry@kau.edu.sa (E.A.E.A.); 2 Restorative Dentistry Department, Faculty of Dentistry, King Abdulaziz University, Jeddah 21589, Saudi Arabia; taabakhsh@kau.edu.sa

**Keywords:** adaptation, pulp- capping, MTA, biodentine, TheraCal, micro–CT, OCT

## Abstract

The objective of this study was to assess and compare the internal adaptation of various pulp-capping materials, namely TheraCal, Biodentine, and mineral trioxide aggregate (MTA), on the dentin of permanent teeth through the utilization of micro-computed tomography (MCT) and optical coherence tomography (OCT). Thirty permanent molars were divided into three groups using a random process: group A (TheraCal), group B (Biodentine), and group C (MTA, which served as the control group). On the buccal surface of each tooth, a class V cavity of a standardized cylindrical shape was prepared. Subsequently, the respective pulp-capping material was applied to the cavity based on the assigned group, followed by restoration with composite resin. Based on the MCT results, it was observed that group A had a considerably larger gap volume in comparison to groups B and C (*p* < 0.001). There was no significant difference in gap volume between groups B and C. Regarding the OCT findings, group A displayed a substantially higher level of light reflection than groups B and C (*p* < 0.001). Group C exhibited a significantly lower level of light reflection in comparison to group B (*p* < 0.001). Biodentine and MTA revealed similar outcomes in terms of how well they adhered to the dentinal surface in permanent teeth. Both materials exhibited superior performance in comparison to TheraCal. The utilization of OCT in clinical practice could be advantageous as it enables dentists to monitor and evaluate restorations during post-treatment follow-up. It is imperative to intensify efforts aimed at making OCT equipment more accessible and applicable, overcoming its current limitations, and allowing for its widespread utilization in clinical practice.

## 1. Introduction

In the early 1990s, an innovative material called mineral trioxide aggregate (MTA) was introduced for the first time as a root-filling material [1]. Nowadays, MTA belongs to a family of pulp-capping agents known as calcium silicate materials (CSMs) [2,3]. The term biocompatibility is often used to describe the capacity of a material to effectively function in a particular application while evoking an appropriate response from the host. This attribute is what makes CSMs highly promising [2,4,5,6]. CSMs have been found to possess high biocompatibility and the ability to induce the formation of dentin bridges. Moreover, they exhibit an outstanding regenerative response [2]. MTA is also able to promote mineralization and repair in the exposed pulp, preserving pulp vitality. Furthermore, MTA is a material that has been extensively researched and used for two decades and has established its biocompatibility and success [1,3,7]. When comparing calcium hydroxide with MTA, it was observed that MTA exhibits a superior response in terms of pulp healing [1]. Furthermore, the clinical trials conducted with MTA as pulp-capping medications demonstrated a high success rate during the follow-up period of up to 4 years [2,3].

Nevertheless, to address issues with extended setting time and high cost, newer modifications and types of MTA have been developed [2,8]. Products like Biodentine, TheraCal, and others have been widely utilized in clinical practice. Biodentine is a cement based on tricalcium silicate and has a setting time ranging from 9 to 12 min [9,10]. Biodentine can seal the margins of the cavity by creating tag-like structures with dentin bond strength similar to that of ProRoot MTA, due to its ability to penetrate the dentinal tubules. It was also found that Biodentine is suitable as a replacement for dentine in the sandwich technique and can be overlaid with a composite [11]. On the other hand, TheraCal is a calcium silicate-based material that is set by light curing and used for indirect and direct pulp-capping procedures. It can also facilitate the immediate application of the final restoration [12]. Yet, few studies have been conducted on Biodentine and TheraCal LC’s internal adaptation to the dentine of permanent teeth when utilized as an indirect pulp-capping material. Moreover, the findings have been inconsistent [9,13,14]. Additionally, a recent review concludes that these new materials require further investigation to better understand their cytotoxicity, bond strength, and dentin-bridge formation quality [15]. Table 1 illustrates some controversy in previous in vitro investigations that examined the efficacy of CSMs in sealing ability.

Conventional radiographs cannot detect the gaps in the internal margins [20]. However, recent innovations, such as micro-computed tomography (MCT) have been used to assess restorations’ internal margin adaptation [19,21]. The difference between it and conventional CT scanners is the voxel size. In CT the volume of the voxel is approximately 1 mm3, while in MCT the voxel size is about 5 to 50 µm, which means it is about one million times smaller [22]. This recent advancement in imaging technology offers the ability to achieve a three-dimensional qualitative and quantitative evaluation of the entire restorative interface in a short time and in more detail [5,6,23,24]. However, radiation hazards and the high cost of the X-ray machine are fundamental limitations [25].

Another recent imaging method is optical coherence tomography (OCT), which has multiple characteristics that make it very promising for various clinical applications [20]. This technique has many merits, including non-invasiveness, visibility of hard and soft tissues, and lack of radiation with excellent spatial resolution [26,27]. OCT allows for real-time imaging of tissue sections without the need for biopsy, histological procedures, or X-rays, in a non-contact and non-invasive manner [28,29,30,31]. The benefits mentioned have been verified in a prior investigation that established the capability of MCT and OCT to identify, without causing damage, the sealing ability of the pulp-capping agents in primary teeth. The examination demonstrated a notable and significant positive association between MCT and OCT [19,20]. However, no studies have compared MCT and OCT’s performance to detect the gap at the cavity floor in permanent teeth. Consequently, this research aims to tackle both concerns through the examination of two aspects: First, the study aims to evaluate the adaptation of recent pulp-capping materials to the dentin surface in permanent teeth; second, it aims to assess these materials using OCT and MCT for a comprehensive and precise evaluation.

## 2. Materials and Methods

### 2.1. Ethical Approval and Sample Size

The protocol for this research obtained ethical approval from the Ethical Committee at the University Dental Hospital of King Abdulaziz University (KAU). The statistical power of the MCT and OCT findings was analyzed to determine the research power. The power of the study to correctly reject the null hypothesis was on the ideal level in both MCT and OCT findings.

### 2.2. Study Sample and Grouping

In total, 30 sound permanent molars were collected, which were freshly extracted for either periodontal or orthodontic reasons. These molars underwent thorough cleaning using ultrasonic scalers and were subsequently kept in distilled water. The molars were then randomly allocated into 3 groups (*n* = 10): group A: TheraCal (Bisco, Anaheim, CA, USA), group B: Biodentine (Septodont, Saint-Maur des-Fossés, France), and group C (the control group): MTA (Dentsply, Charlotte, NC, USA). The randomization process was conducted using SPSS v. 20.0 (IBM, Armonk, NY, USA) for Windows.

### 2.3. Preparation of Specimens

A skilled operator prepared a standardized cavity in the cervical region of each tooth, utilizing a milling machine equipped with a constant stream of water to cool the cavity during the process. To ensure optimal performance, the operator replaced each bur after completing 5 cavities. The prepared cavities had a depth of 2 mm and a width of 3 mm, with the cavity floor resting on dentine.

### 2.4. Preparation and Application of the Different Pulp-Capping Materials

In Group A, the material was administered into the cavity using a syringe tip and cured for 20 s using light in accordance with the manufacturer’s guidelines. In Groups B and C, the materials were mixed as per the manufacturer’s instructions, and the pulp-capping material was applied using an amalgam carrier with a small tip. Subsequently, condensation was conducted using an amalgam condenser. According to the manufacturer’s instructions, TheraCal sets shortly after light curing, specifically after 20 s.

### 2.5. Application of Restorative Materials

To perform the cavity restoration, the fifth-generation bonding material (Single Bond, Adper-3M-ESPE, USA). The composite restoration (Filtek-Z350-XT, 3M-ESPE, USA) was then applied in incremental layers using light-curing device (Elipar-Curing Light-2500, 3M-EPSE, USA). Following that, all restorations in the sample were cured utilizing the same light-curing device. To simulate natural oral conditions, the samples underwent 1000 cycles of thermocycling (5–55 °C) with a dwell time of 20 s using a thermocycling device (SD Mechatronic Machine, GmbH comp., Germany).

### 2.6. Evaluation by MCT (Scanning and Analysis)

The Skyscan Model 1172 device, produced in Kontich, Belgium, was utilized for MCT scanning in this study. To reconstruct the generated images, NRecon v. 1.6 software was used, which is specifically designed for this purpose. To ensure accurate and stable scanning, each specimen was carefully affixed to a specimen holder and secured with wax. This step was crucial to maintain stability throughout the MCT scanning process, minimizing any potential movement or distortion.

In this study, the measurements were conducted in mm3 for precise quantification of gap volume and total restorative volume. To delineate the specific region of interest, CT Analyzer software v. 1.11 was used. The region of interest was delineated between the dentine surface and the pulp-capping material. Subsequently, the specimens were further segmented into CT slices based on their grayscale values. This segmentation process enabled us to accurately determine the volumes of both the gap and the restoration. The volume of the gap was calculated by inverting the segmented image in the area of interest according to shade density. A one-click process was used to analyze the images obtained in the region of interest in the evaluation software.

### 2.7. Evaluation by OCT (Scanning and Analysis)

A portable cross-polarization OCT scanner (CP OCT, IVS 300, Japan) was utilized in this investigation. The system was connected to a handheld diode laser probe with a scanning rate of 30 kHz. The laser had a continuous wavelength of 1310 nm and a wavelength range of 100 nm. The device’s sensitivity is 95 dB, and the lateral and axial resolution were 30 and 12 μm, respectively. The energy output of the probe adhered to the safety guidelines established by the American National Standards Institute. The sample was secured in place on a micrometer stage, and then serial tomographic B-scans were taken to evaluate the gaps in each 250 μm [32]. The scans obtained from OCT were moved to an external drive and then re-evaluated by an additional personal computer with image analysis software (ImageJ v. 1.5m9, National Institutes of Health, Bethesda, MD, USA). The images obtained were then reconstructed in grayscale using a previously described protocol [32]. The floor of the cavity was determined, and then measured, and binary pictures of black and white pixels were obtained. The regions with increased backscattered reflections on a background of white were transformed into black pixels, which indicated the gaps. The percentage of gaps on the restoration’s floor was evaluated using the following equation:

$$Gap\;percentage=\left(\frac{Gap\;width}{Width\;of\;the\;cavity\;floor}\right)\times 100$$

### 2.8. Statistical Methods

The data collected in this study were analyzed using SPSS version 20.0 for Windows. A significance level of *p* < 0.05 and a 95% confidence level were selected. To assess the normality of the data, the Shapiro–Wilk test was conducted. The test yielded a *p*-value of 0.48, indicating that the data followed a normal distribution. Based on this finding, a one-way analysis of variance (ANOVA) was chosen to compare the results of MCT and OCT.

In order to conduct a more thorough examination of the relation between the findings from MCT and OCT, a Pearson correlation test was employed. This test enabled the assessment of both the degree and direction of the correlation. To determine the extent and intensity of the correlation, the method proposed by Akoglu and Haldu in 2018 was implemented [33]. This method established a threshold of r = pm 0.7 to categorize correlations as strong to moderate, and r = pm 0.4 to classify correlations as moderate to weak.

## 3. Results

### 3.1. MCT Findings

One-way analysis of variance (ANOVA) was utilized to evaluate the MCT findings. The examination of the total cavity volume revealed means of 10.875 ± 1.29, 10.888 ± 1.28, and 10.892 ± 1.27 for groups A, B, and C, respectively. However, none of these differences were deemed significant (*p* = 1). For the total gap volume, group A displayed a mean of 0.253 ± 0.03, group B had 0.035 ± 0.03, and group C had 0.027 ± 0.027. The differences observed between these groups, however, were found to be significant (*p* < 0.001). Similarly, concerning the mean ratio of the gap to total cavity volume, group A exhibited a mean ratio of 2.352 ± 0.388, group B had 0.317 ± 0.270, and group C displayed 0.253 ± 0.275. Again, the differences between these groups were noted as significant (*p* < 0.001).

To further investigate the differences between the groups, the LSD test was employed, and the results are presented in Table 1. Group A demonstrated significantly greater gap volume than both groups B and C (*p* < 0.001). On the other hand, no significant difference in total gap volume was observed between groups B and C (*p* = 0.59). 

**Figure 1:**
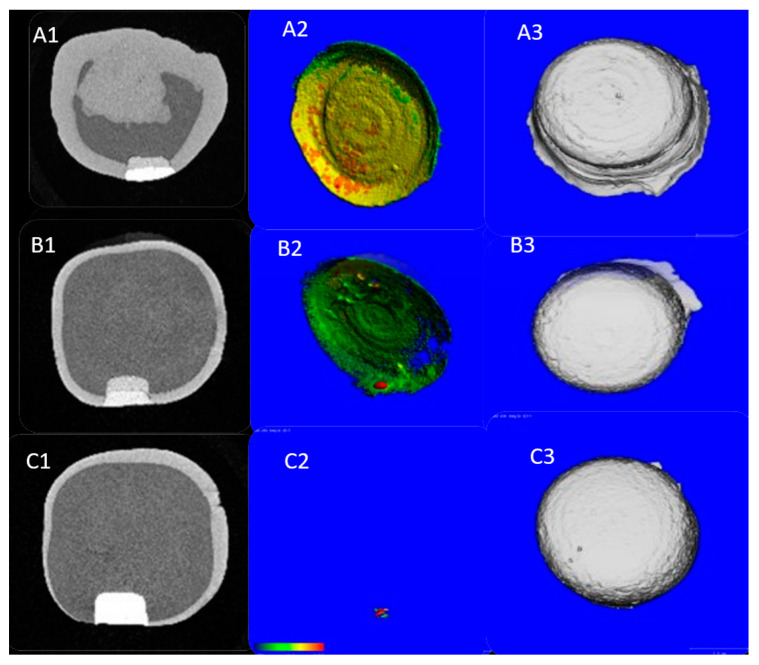
The MCT images for all groups are as follows: (**A**) TheraCal, (**B**) Biodentine, and (**C**) MTA. The first column (A1-B1-C1) contains 2D cross-sectional views of the samples, while the second column (A2-B2-C2) displays the gap’s thickness within the region of interest, color-coded from blue (thinnest) to red (thickest). The third column (A3-B3-C3) shows the 3D renderings of the samples, including the filing and pulp-capping materials for each group.

In terms of the mean ratio of gap to total restorative volume, group A exhibited a significantly higher gap volume compared to groups B and C (*p* < 0.001), while the total gap volume in groups B and C did not vary significantly (*p* = 0.66). Table 2 and Figure 1 provide a summary of the mean differences between groups.

### 3.2. OCT Findings

The scans of OCT were analyzed to assess the intensity of high reflection at the interface between pulp capping material and dentinal surface for each specimen. The one-way ANOVA test was used to test the findings form OCT in permanent teeth.

**Figure 2:**
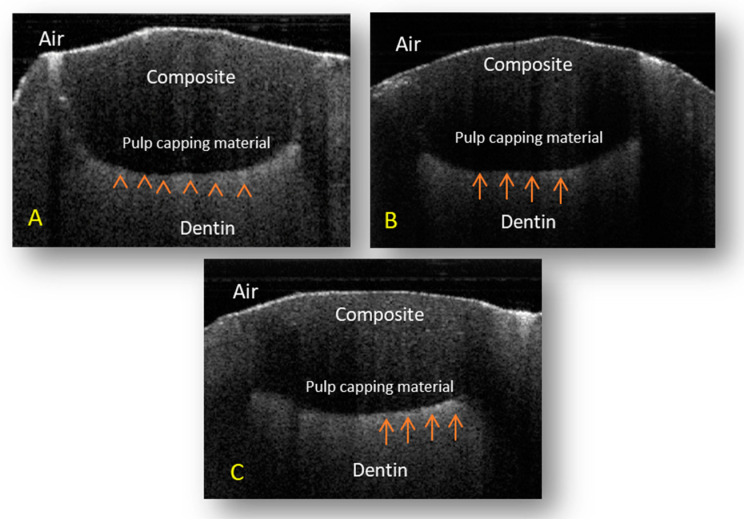
Representative images were taken for the tested groups, with TheraCal shown in (**A**), Biodentine shown in (**B**) and MTA shown in (**C**). Composite restorations and the interface between the pulp- capping material and the dentinal surface can be seen in each scan. The floor of the cavity with high light reflection is indicated by red arrows.

A summary of the results and mean differences between the groups is shown in Table 3, and the scans of OCT for the groups are shown in Figure 2. All groups showed some degree of loss of the interfacial seal in the form of bright pixels that presented as high-intensity reflection (HIR), while areas that did not show a loss of the interfacial seal presented as dark pixels. 

The mean percentage of HIR on the floor in the groups differed significantly. That of group A was 86.54 ± SD = 1.97, that of group B was 82.06 ± SD = 2.19, and that of group C was 77.29 ± SD = 3.82 (*p* < 0.001). The LSD test was used to assess the differences between the three groups. Significantly higher HIR was observed in group A compared to all groups (*p* < 0.001), while group B had significantly higher HIR than group C (*p* < 0.001).

### 3.3. Comparative Analysis of Findings from MCT and OCT

**Figure 3:**
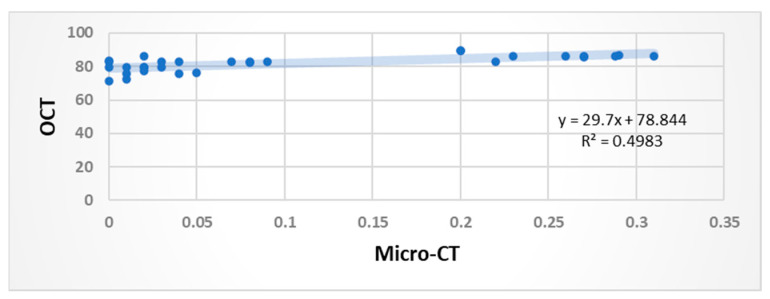
As the scatter plots indicate, a significant positive correlation of r = 0.70 was observed between the MCT and OCT findings.

There A strong positive correlation was observed between the abilities of MCT and OCT to assess the quality of adaptation in permanent teeth. This was confirmed by the significant Pearson correlation coefficient of 0.71, which also indicated that higher MCT values were associated with higher OCT values. A Pearson’s correlation test was conducted to establish the relationship between the findings from MCT and OCT, as shown in Figure 3. The test yielded a result of r = 0.706, *n* = 30, *p* < 0.001.

## 4. Discussion

Several techniques are used in dentistry to assess different types of restorative materials’ sealing ability and leakage resistance. These techniques include radioisotope tracers, bacterial leakage models, electrochemical methods, fluids infiltration, and penetration of the dye [34]. Unfortunately, these techniques require slicing to evaluate leakage and thus destroy the samples. Also, this method can be subjective since the dye can stain the adjacent sound dentine, leading to false positive findings [21,35,36]. Among the contemporary assessment methods are MCT and OCT, the advantages of these methods include the no destruction of the sample when evaluating the restorations’ internal adaptation [34]. Further, MCT and OCT can conveniently detect the microgaps and areas with defects in the restoration’s internal construction in two and three dimensions [36,37]. These are the primary reasons to implement the two imaging methods to assess pulp-capping agents’ internal adaptation.

The study maintained consistent methods for teeth, cavities, storage, and restoration. The operator used a milling machine to standardize the restorative procedure and prepare all the samples. This was reflected in the dimensions of the prepared cavities in the MCT assessment, which showed no dimensional discrepancies upon evaluating the restoration volume for all groups tested. Moreover, each pulp-capping material’s setting time was estimated after reviewing previous reports and the manufacturer’s recommendations (6). Then, the pulp-capping agents were covered with a cotton pellet to ensure that the materials were appropriately set by supplying optimal hydration for the setting reaction before the final restoration was applied. According to previous studies [9,38], the hydration reaction times required for MTA and Biodentine to set completely are 12 min and 4 h, respectively, while according to the manufacturer’s instructions, TheraCal sets immediately after light curing (20 s). Regarding the composite, the dental nanocomposite that was used in the current study has shown in previous research similar levels of translucency, polish, and polish retention as microfills, while also maintaining the same physical properties and wear resistance as several hybrid composites [39].

Based on the MCT and OCT findings in this work, comparable outcomes were observed between Biodentine and MTA in the sealing ability, although to some extent, MTA demonstrated better performance than Biodentine. This was consistent with other studies [13,14,40,41,42], which found that MTA and Biodentine showed similar adaptation. MTA is expected to exhibit excellent adaptation is likely attributable to its expansion over time while it sets, which may reduce the gap within the cavity [43]. Moreover, this material’s success may be attributable to its inherent ability to release calcium hydroxide and its ability to seal the dentine [44].

The findings pertaining to MTA and Biodentine yielded inconsistent results in comparison to other studies [16,45,46]. However, this discrepancy may be attributed to variations in the procedural testing of adaptation and sealing. Studies may have utilized a variety of techniques, such as utilizing these materials for the simulation of pulp exposure, for repairing perforations, or as an apical seal. Moreover, distinct setting environments may play a role in producing different results in terms of their effectiveness in establishing a seal.

The current study found that TheraCal had a higher internal gap at the dentinal surface compared to MTA and Biodentine. Nevertheless, a laboratory investigation utilizing laser confocal microscopy revealed that TheraCal demonstrated superior sealing performance compared to MTA and Biodentine, which contradicts the results of this study [18]. It has been documented that TheraCal should be applied directly on the dentine surface without any bonding adhesive and then cured according to the manufacturer’s instructions [47]. Adding 43% resin to the TheraCal without using any bonding adhesive may affect the material’s appropriate adaptation to the dentine surface. Earlier research has verified these findings, examining the bonding strength of this material to dentin surfaces both with and without the use of adhesive bonding. TheraCal’s shear bond strength increased significantly to 11.1 MPa with bonding adhesive and decreased to 0.8 MPa when the dental adhesive step was omitted [19,48]. Moreover, Camilleri et al. found that TheraCal LC did not hydrate completely because of improper diffusion of water derived from the pulp–dentine complex within this material [49]. Thus, it can be concluded that the resin composition and inadequate setting of this material may contribute to its poor adaptation [19,48,49].

Both OCT and MCT have distinct features for assessment. MCT can evaluate restorations without being limited by specific cavity depth. However, its use to assess dental restorations in humans is considered unethical and unjustified. On the contrary, OCT is not based on radiation, and in the future, OCT could be a potential substitute for dental X-ray images for clinical evaluation of the dental restorative materials’ performance hence avoiding the biological effects of the radiation. However, it has imaging depth limitations and cannot be used for deep cavities or bulky restorations [50].

The significant correlation between the results obtained from MCT and OCT is consistent with prior research investigations. It was proven through the Pearson correlation coefficients that high values in MCT correspond to high values in OCT [21,51,52]. However, it is important to note that both MCT and OCT have their characteristic features that make them valuable methods for evaluation. Both imaging systems can generate micron-resolution images. However, unlike OCT, MCT has no limitation in the cavity depth that can be evaluated, whereas OCT can reveal a clear image of the target area at the micron scale, unlike MCT. OCT cannot be used in deep cavities or bulky dental restorations as it has imaging depth limitations. Fortunately, this was not problematic in this study because of the utilization of a relatively shallow cavity (only 2 mm deep), while the acceptable depth for using OCT was reported to be 0.5–3 mm and varied according to the material’s index of refraction [50].

This study is acknowledged for being one of the limited investigations that concurrently analyze the sealing ability of three advanced calcium silicate-based pulp- capping materials as agents for indirect pulp- capping. Furthermore, this study establishes a correlation between OCT and MCT in evaluating the sealing ability of pulp-capping materials. Traditional evaluation methods, such as tooth sectioning, have unfortunately resulted in the destruction of specimens [25,53]. To overcome this limitation, non-destructive state-of-the-art assessment techniques, specifically MCT and OCT were used.

Dental research often requires extensive empirical studies involving numerous participants, which can be time-consuming, expensive, and ethically challenging. In silico simulation offers a promising alternative, allowing researchers to virtually conduct experiments and investigate the performance of new dental materials, devices, and procedures without physically subjecting patients to invasive interventions [54,55,56,57,58,59,60,61,62,63]. The current study has the potential to be conducted using an in silico approach. By allowing for virtual modeling and testing, in silico simulation enables dentists, researchers, and manufacturers to explore and optimize various dental interventions, techniques, and materials. Simulated computer models can assist in detecting subtle anomalies or irregularities that may otherwise be overlooked [54,55,56,57,58,59,60,61,62,63]. Furthermore, complex biological processes can be simulated and analyzed in a virtual environment, thus eliminating the necessity for time-consuming and costly laboratory-based experiments [54,55,56,57,58,59,60,61]. This method allows us to conduct large-scale simulations and data analysis, resulting in faster and more accurate results. Given the advancements in technology and the fast-paced nature of the scientific field, it is highly recommended to explore the use of in silico methods for similar research.

This study has some limitations. When interpreting these results, other factors not examined in the current study that could affect the success of any pulp-capping agent should be considered (e.g., bioactivity. Furthermore, the sample size of the study was small, which was attributed to the difficulty in obtaining intact molar teeth. Another limitation of this study is using a cavity on the buccal surface rather than the occlusal surface. Even with the reliability of the assessment methods used in this study to evaluate the pulp-capping agents’ adaptation, the results still need to be confirmed using conventional methods of assessment and clinical trials.

## 5. Conclusions and Recommendations

It can be concluded that Biodentine and MTA exhibited more promising outcomes in adaptation than TheraCal. OCT is a promising technology. However, it is imperative to intensify efforts aimed at making OCT equipment more accessible and applicable, overcoming its current limitations, and allowing for its widespread utilization in clinical practice. By ensuring affordability, addressing technological constraints, fostering interdisciplinary collaboration, and providing comprehensive training, the integration of OCT technology in routine clinical settings can be significantly enhanced. This will subsequently lead to improved patient care, diagnostic accuracy, and treatment outcomes across a multitude of healthcare specializations.

## Figures and Tables

**Table 1:** Instances of previous in vitro investigations examining the efficacy of CSMs in sealing ability.

Study	Main Findings	Remarks
Ravichandra et al., (2014) [16]	Biodentine showed superior adaptation when compared with MTA.	Utilized as root filling materials.
Sulaiman et al., (2014) [17]	Biodentine and MTA demonstrated comparable outcomes in terms of adaptation when used as restoration materials.	The assessment was based on sectioning of the specimens and dye staining.
Makkar et al., (2015) [18]	TheraCal exhibited superior sealing and reduced leakage compared to MTA and Biodentine.	The assessment was based on one method (confocal laser microscopy).
Al Tuwirqi et al. (2021) [19]	Biodentine and MTA exhibited comparable outcomes to the dentinal surface of primary teeth, outperforming TheraCal.	Conducted in primary teeth.

**Table 2:** One-way ANOVA and LSD test for the MCT findings.

Dependent Variables	Group	*n*	Mean (mm^3^)	SD	SE	*p* Value	LSD	
							Group	*p*-Value
Total restorative volume	A	10	10.875	1.297	1.297	1.00	A–B	1.00
	B	10	10.888	1.283	1.283		A–C	1.00
	C	10	10.892	1.279	1.279		C–B	1.00
Total gap volume	A	10	0.253	0.039	0.039	<0.001 *	A–B	<0.001 *
	B	10	0.035	0.031	0.031		A–C	<0.001 *
	C	10	0.027	0.027	0.027		C–B	0.592
Ratio of gap to total restorative volume	A	10	2.352	0.388	0.388	<0.001 *	A–B	<0.001 *
	B	10	0.317	0.270	0.270		A–C	<0.001 *
	C	10	0.253	0.275	0.275		C–B	0.655

*: Statistically Significant *p* < 0.05. A: TheraCal, B: Biodentine, and C: MTA. LSD: Least significant difference test.

**Table 3:** One-way ANOVA and LSD test for the OCT findings.

Group	N	Mean Percentage	SD	SE	*p* Value	LSD	
						Group	*p* Value
A	10	86.54	1.97	0.62	<0.001 *	A–B	<0.001 *
B	10	82.06	2.19	0.69		A–C	<0.001 *
C	10	77.29	3.82	1.21		B–C	<0.001 *

*: Statistically significant *p* < 0.05. A: TheraCal, B: Biodentine, and C: MTA. LSD: Least significant difference test.

## Data Availability

The data presented in this study are available on request from the corresponding author.
